# Participation des médecins généralistes de la province de Benimellal (Maroc) dans le dépistage du cancer du col

**DOI:** 10.11604/pamj.2013.14.152.2570

**Published:** 2013-04-17

**Authors:** Samira Nani, Mohamed Benallal, Samira Hassoune, Dounia Kissi, Abderrahmane Maaroufi

**Affiliations:** 1 Laboratoire d'Epidémiologie-Faculté de Médecine et de Pharmacie de Casablanca, Maroc; 2 Médecin généraliste, Casablanca, Maroc; 3 Médecin responsable du Programme Cancer au Service de santé publique et de surveillance épidémiologique-Direction régionale de la santé du Grand Casablanca, Maroc

**Keywords:** Cancer du col utérin, médecin généraliste, soins de santé de base, dépistage, Cervical Cancer, general practitioner, basic health care, screening

## Abstract

**Introduction:**

Au Maroc, chaque année il y aurait environ 2000 nouveaux cas de cancer du col et les 2/3 des cas sont pris en charge à un stade très avancé. Nous avons mené une étude transversale, exhaustive incluant les 71 médecins généralistes exerçant dans les établissements de soins de santé de base du secteur public et privé de la province de Benimellal. Le but était d’évaluer leurs connaissances et leur participation au dépistage du cancer du col.

**Méthodes:**

Nous avons mené une étude transversale, exhaustive incluant les 71 médecins généralistes exerçant dans les établissements de soins de santé de base du secteur public et privé de la province de Benimellal. Le but était d’évaluer leurs connaissances et leur participation au dépistage du cancer du col.

**Résultats:**

Le niveau de connaissance était relativement modeste, 22 médecins généraliste avaient répondu à la question sur l'incidence du cancer du col au Maroc, Parmi eux (81,8%) avaient donné une réponse incorrecte. L'Herpes Papilloma virus comme facteur de risque du cancer du col a été identifié par seulement 21% des médecins généralistes. La participation au dépistage était également défaillante, 92,8% n'avaient jamais pratiqué le FCV chez leurs patientes à cause principalement du manque de formation (95,5%).

**Conclusion:**

Les résultats montrent la nécessité d'améliorer les connaissances théoriques et pratique des médecins généralistes concernant le dépistage du cancer du col.

## Introduction

A l’échelle mondiale, le cancer du col utérin est le deuxième cancer le plus fréquent chez la femme après le cancer du sein. Selon l'OMS, Le cancer du col de l'utérus a provoqué, en 2005, près de 260 000 décès dont près de 95% dans les pays en voie de développement, pays dans lesquels ce cancer est la première cause de mortalité par cancer dans la population féminine [1]. En l'absence d'intervention rapide, la mortalité associée au cancer du col augmenterait de 25% dans les dix prochaines années. Ces décès peuvent être évités car le cancer du col est l'un des cancers les plus faciles à prévenir et à traiter à condition qu'il soit détecté suffisamment tôt et traité correctement [1].

Au Maroc, d'après les résultats du registre du cancer de Casablanca au Maroc, l'incidence du cancer du col était de 14 pour 100 000 femmes en 2007 [2] et au niveau national chaque année il y aurait environ 2000 nouveaux cas de cancer du col et les 2/3 des cas de cancer sont diagnostiqués et pris en charge à un stade très avancé dans les différents centres d'oncologie [3]. Depuis 2010, le Maroc a instauré un programme de dépistage du cancer du col et du sein qui sera intégré dans les activités de santé de la reproduction et mis en place progressivement au niveau national. Ce programme est inclus dans la cadre du programme national de prévention et de contrôle des cancers (PNPCC). La province de Beniméllal ne fait pas partie des sites pilotes et sera prochainement intégré dans ce programme [3]. L'importance du rôle du médecin généraliste dans le dépistage du cancer du col a été largement démontrée par plusieurs auteurs [4, 5]. En raison de leur place central dans le système de soin et de leur rôle primordial en matière d'information et d'orientation des patients; les médecins généralistes peuvent contribuer efficacement à la sensibilisation, l'information et la motivation des femmes vis-à-vis du dépistage [4–6]. La prévention passe obligatoirement par des connaissances et des pratiques adéquates des médecins généralistes concernant la maladie et son dépistage.

L'objectif de notre travail était d’évaluer les connaissances du médecin généraliste sur le cancer du col et son dépistage, de connaitre le degré de participation pratique du médecin généraliste dans le dépistage du cancer du col dans la province de Benimellal au Maroc.

## Méthodes

Une étude transversale descriptive a été conduite chez les médecins généralistes de la province de Benimellal, au Maroc. La population totale de cette région a été estimée en 2012 à 500 594 habitants dont 257 579 en milieu urbain et 243 015 en milieu rural. Cette province est dotée de 45 établissements de soins de santé de base publics dont 13 en milieu urbain et 32 en milieu rural, un hôpital régional de Benimellal et un hôpital local de Kasbat Tadla. Ont été inclus tous les 81 médecins généralistes exerçant dans les établissements de soins de santé de base du secteur public (35) et dans les cabinets privés (46). Le consentement éclairé des médecins a été obtenu avant leur inclusion dans l’étude. Par ailleurs, l'anonymat et le respect de la confidentialité des données ont été assurés et l'accord du comité d’éthique n'a pas été nécessaire car ce dernier ne donne son avis que sur les études interventionnelles. L'enquête s'est déroulée d'Aout 2012 à Novembre 2012. La collecte des données a été faite par un questionnaire auto administré préalablement testé, il incluait les caractéristiques sociodémographiques et professionnelles, les connaissances sur le cancer du col et son dépistage par frottis cervico-vaginal (FCV), les pratiques personnelles et professionnelles vis-à-vis du dépistage du cancer du col. Ont été considérés comme réponse correcte, une incidence comprise entre 10 et 15 pour 100000 et un intervalle de dépistage de trois ans effectué entre 25 et 65 ans selon les critères de l'OMS [1]. Les données ont été saisies et analysées grâce au logiciel SPSS version 16 sous Windows. On a calculé la moyenne et l’écart-type pour les données quantitatives et les proportions pour les variables qualitatives. Nous avons utilisé le test de Khi2 pour la comparaison des pourcentages, le seuil de signification a été fixé à 5%.

## Résultats

Sur les 81 médecins ciblés par notre étude 71 médecins ont répondu au questionnaire, soit un taux de participation de 87%. Le taux de participation était plus élevé dans le secteur public (91,4%) par rapport au secteur privé (84.8%). Le manque de temps était le motif de non-participation le plus évoqué. L’âge moyen des médecins était de 44,22 ans plusmn; 7,02 ans, 65,7% étaient de genre masculin. L'ancienneté d'exercice moyenne était de 14,4 ans plusmn; 6,7 ans. Les autres caractéristiques socioprofessionnelles des médecins sont résumées dans le Tableau 1. Concernant les connaissances sur le cancer du col, uniquement 22 (31%) ont répondu à la question sur l'incidence, parmi eux la plupart (81,8%) avaient donné une réponse incorrecte. La plupart des médecins interrogés 96,9% avaient mentionné que la survie au cancer du col au stade infra clinique dépasse les 80%. Presque tous les médecins (94,4%) savaient que le cancer du col était curable si détection précoce. Concernant les facteurs de risque du cancer du col, le facteur de risque le plus mentionné était les infections sexuellement transmissibles (77,5%). Alors que seulement 21,1% savaient que l'herpes papilloma virus (HPV) était un facteur de risque du cancer du col, les autres facteurs mentionnés étaient le tabac, les rapports sexuels précoces, les partenaires sexuels multiples, la contraception, la multiparité, l'infection HIV et l'immunodépression cités respectivement par 29,6%, 25,4%, 21,1%, 16,9%, 16,9%, 5,6% des médecins généralistes. Aucun des médecins généralistes n'a révélé le bas niveau socio-économique comme facteur de risque du cancer du col (Tableau 2).

**Tableau 1:** Caractéristiques socioprofessionnelles des médecins généralistes de la province de Benimellal, Maroc

Pratiques du dépistage du FCV	n	%
Médecins généralistes ayant appris à faire le dépistage par FCV		
(n = 71)	4	5,6
Recevoir souvent des femmes dans la consultation (n = 71)	70	98,6
Faire fréquemment (toujours, souvent) un examen vaginal des femmes (n = 71)	34	47,9
Utilisation du speculum (toujours, souvent) lors de l'examen vaginal (n = 34)	24	70,6
Demande aux femmes si elles ont déjà pratiqué un dépistage du cancer du col (n = 71)	55	77,5
Référence des femmes pour dépistage du cancer du col (n = 71)	45	63,4
Médecins pratiquant le dépistage du FCV chez leurs patientes (n = 69)	5	7,2
Médecins généralistes ayant bénéficié elles-mêmes (femmes) ou leurs partenaires (hommes) de FCV (n = 71)	18	25,4
Médecins généralistes femmes ayant bénéficié elles-mêmes de FCV (n = 43)	9	20,9
Médecins généralistes hommes dont les partenaires avaient bénéficié de FCV (n = 28)	9	32,1

**Tableau 2:** Connaissances des médecins généralistes de la province de Benimellal (Maroc) concernant le cancer du col

Connaissances	n	%
Incidence (n = 22)	4	18,2
Survie à 5 ans au stade infra clinique ≥ 80% (n = 64)	62	96,9
Curable si détection précoce (n= 71)	69	97,3
Début du dépistage à l’âge de 25 ans (n = 69)	2	2,9
Arrêt du dépistage à l’âge de 65 ans (n = 68).	0	0
Intervalle du dépistage tous les 3 ans (n = 70)	32	45,7
Connaissance de l'HPV comme facteur de risque (n = 71)	15	21,1

Concernant les femmes à dépister par FCV, seulement 2,9% savaient que le dépistage doit débuter à l’âge de 25 ans et 47,1% jugeaient qu'il fallait le poursuivre jusqu’à l’âge de 45 ans et aucun médecin n'avait jugé qu'il fallait le poursuivre jusqu’à l’âge de 65 ans. Un intervalle de dépistage de1 fois tous les 3 ans était donné par 45,7% des médecins généralistes, 37,1% et 15, 7% recommandaient un rythme plus court respectivement 1 fois tous les 2ans et 1 fois par an. Presque tous les médecins (98,6%) recevaient souvent des femmes dans leurs consultations et uniquement 5,6% avaient déjà reçu une formation sur la pratique du FCV.

Lors de leur pratique quotidienne, 47,9% des médecins généralistes pratiquaient souvent ou toujours un examen vaginal des femmes, 67,9% utilisaient souvent ou toujours un speculum lors de l'examen vaginal. Les médecins exerçant dans le milieu urbain faisaient plus l'examen vaginal des femmes (55%) que les médecins exerçant dans le milieu rural (38,5%), cette différence n’était pas statistiquement significative (P = 0,232). Egalement, les médecins femmes avaient tendance à faire plus l'examen vaginal aux femmes (50%) par rapport aux médecins hommes (46,5%). Cette différence n’était pas statistiquement significative (P = 0,812). La plupart des médecins généralistes (77,5%) demandaient souvent ou toujours à leurs patientes si elles ont pratiqué un frottis de dépistage. Les médecins du secteur privé demandaient plus à leurs patientes si elles ont déjà pratiqué un dépistage du cancer du col (79,5%) par rapport aux médecins du secteur public (75%), cette différence n’était pas statistiquement significative (P = 0,777). Plus de la moitié des médecins généralistes, 63,4% référaient souvent ou toujours leurs patientes pour dépistage. Les médecins exerçant dans le milieu urbain referaient plus les femmes pour dépistage du cancer du col, que les médecins exerçant dans le milieu rural (60%), cette différence n’était pas statistiquement significative (P = 0,621) (Tableau 3).

**Tableau 3:** Pratiques liées au dépistage du cancer du col chez les médecins généralistes de la province de Benimellal, (Maroc)

Pratiques du dépistage du FCV	n	%
Médecins généralistes ayant appris à faire le dépistage par FCV (n = 71)	4	5,6
Recevoir souvent des femmes dans la consultation (n = 71)	70	98,6
Faire fréquemment (toujours, souvent) un examen vaginal des femmes (n = 71)	34	47,9
Utilisation du speculum (toujours, souvent) lors de l'examen vaginal (n = 34)	24	70,6
Demande aux femmes si elles ont déjà pratiqué un dépistage du cancer du col (n = 71)	55	77,5
Référence des femmes pour dépistage du cancer du col (n = 71)	45	63,4
Médecins pratiquant le dépistage du FCV chez leurs patientes (n = 69)	5	7,2
Médecins généralistes ayant bénéficié elles-mêmes (femmes) ou leurs partenaires (hommes) de FCV (n = 71)	18	25,4
Médecins généralistes femmes ayant bénéficié elles-mêmes de FCV (n = 43)	9	20,9
Médecins généralistes hommes dont les partenaires avaient bénéficié de FCV (n = 28)	9	32,1

Les médecins hommes referaient plus leurs patientes pour dépistage par FCV (67,4%) par rapport aux médecins femmes (57,1%). Cette différence n’était pas statistiquement significative (P = 0,453). Tous les médecins interrogés pensaient qu'un médecin généraliste doit apprendre à pratiquer le FCV mais la majorité d'entre eux 92,8% ne l'avaient jamais pratiqué chez leurs patientes. La principale raison de non pratique du FCV chez leurs patientes était le manque de formation cité par 95,5% des médecins généralistes, suivis du manque d'outils pour pratiquer le FCV cité par 32,8% des médecins généralistes. Concernant leur pratique personnelle du FCV, 67,9% des médecins généralistes femmes n'avaient jamais bénéficié elles-mêmes d'un FCV et 79,1% des partenaires des médecins hommes n'avaient jamais fait de FCV. Cette différence n’était pas statistiquement significative (p = 0,403).

Les médecins femmes n'avaient jamais bénéficié de FCV parce qu'elles ne se sentaient pas à risque (55,6%), car elles n'avaient pas de symptômes (33,3%) et parce qu'elles avaient peur de faire un FCV (11,1%). Les médecins hommes dont les partenaires n'avaient jamais bénéficié de FCV, évoquaient comme raisons l'absence de risque (53,1%), l'absence de symptômes (37,5%), l'embarras (6,3%) et la peur de faire un FCV (3,1%).

## Discussion

Notre étude est la première réalisée, selon nos connaissances au niveau de la province de Benimellal, elle est exhaustive incluant 81 médecins généralistes public et privé avec un taux de réponse élevé de 87%. Mais nos résultats doivent être nuancés car il s'agit de déclarations et non de pratiques observées, et il peut exister une différence entre ce qui est dit et ce qui est réellement fait par les médecins.

On a constaté un manque de connaissance concernant l’épidémiologie du cancer du col, en effet seulement 18,2% des 22 médecins généralistes qui ont répondu à la question sur l'incidence ont donné une réponse correcte. Même constat dans une étude tunisienne ou l'incidence n'a été connu que par 11,3% des médecins interrogés [7]. Par contre la plupart des médecins généralistes dans notre étude (96,9%) avait répondu que la survie du cancer du col au stade infra-clinique dépasse les 80%, même résultat dans l’étude tunisienne ou 85,1% des médecins généralistes avait une bonne connaissance de la survie du cancer du col [7]. Presque tous les médecins (94,4%) savaient que le cancer du col était curable si détection précoce, un pourcentage aussi élevé (81,6%) a été retrouvé dans une étude thaïlandaise [8].

L'HPV comme principal facteur de risque du cancer du col a été identifié par seulement 21% des médecins généralistes, un pourcentage aussi faible a été retrouvé dans une étude Ougandaise 29% [9]. D'autres études ont retrouvé un pourcentage plus élevé, étude saoudienne (68,5%) [10] et étude mexicaine (79%) [11]. Alors que depuis les années 90, il est largement documenté que l'HPV est le principal facteur de risque du cancer du col [12].

Aucun des médecins généralistes n'a révélé le bas niveau socio-économique comme facteur de risque du cancer du col. Alors que les femmes ayant un faible revenu sont plus à risque d’être atteintes du cancer du col de l'utérus, surtout parce qu'elles sont moins susceptibles de faire régulièrement le dépistage [13]. Les travaux menés aux USA montrent que chez les femmes les plus pauvres ou issues de minorités, le cancer du col de l'utérus a tendance à être diagnostiquée à des stades plus tardifs et le taux de mortalité à être plus élevé [14].

Concernant les connaissances par rapport au dépistage par FCV, selon les recommandations de l'OMS, le dépistage par FCV doit être fait entre 25 et 65 ans, seulement 2,9% des MG savaient que Le FCV doit débuter à l’âge de 25 ans, dans une étude française un pourcentage bas a été également retrouvé 11% [6].

L'intervalle du dépistage selon les recommandations de l'OMS doit être de 3 ans, cet intervalle a été correctement identifié par 47,5% des MG dans notre étude ce qui rejoint les résultats de l’étude française [6] et Ougandaise [9] ou cet intervalle a été identifié par respectivement 50% et 39% des MG.

Dans une étude française, Plus de 90% des médecins commencent le dépistage par FCV après le début des rapports sexuels ou de la contraception de leurs patientes, 53% arrêtent de pratiquer le frottis entre 60 et 70 ans, seulement 70% suivaient les consignes qui préconisent un frottis tous les 3 ans [15]. Par contre dans une étude mexicaine, 77% des médecins pensent que le FCV doit débuter après le début des rapports sexuels, 10% des Médecins généralistes pensent que les femmes devraient être dépistée après la naissance de leur premier enfant et l'intervalle d'une fois par an a été recommandé par 73% des médecins [11].

Actuellement il n'y a pas encore de consensus international sur l’âge de début ni sur la périodicité du dépistage mais tous s'accordent que le choix doit prendre en compte les moyens de chaque pays et la plupart recommandent une périodicité de 3 ans. Par exemple pour la « US Preventive services Task force » le dépistage par FCV doit être fait entre 21 et 65 ans tous les 3 ans [16].

En France La HAS « la haute autorité sanitaire » recommande le dépistage par frottis cervico-vaginal tous les 3 ans à partir de 25 ans et jusqu’à 65 ans [17].

Au Maroc, un grand effort a été fourni pour mettre en place un système organisé de dépistage, de diagnostic précoce et de traitement du cancer du col dans le cadre du PNPCC [3].

Notre étude nous a donné un état des lieux sur les connaissances des médecins généralistes dans la province avant l'implantation du programme national de dépistage et peut aider les décideurs à mettre en évidence les défaillances à considérer pour garantir la réussite de leur action.

La participation au dépistage était très faible chez les médecins généralistes. En effet, seulement 30% des médecins généralistes femmes avaient déjà bénéficié elle mêmes de FCV et uniquement 19,4% des partenaires des MG hommes ont déjà bénéficié de FCV. Ces pourcentages restent très en dessous de ceux retrouvés dans la littérature, dans une étude ougandaise 81% des praticiens femmes ont déjà bénéficié de FCV et 74% des partenaires des praticiens hommes ont déjà bénéficié de FCV [9].

Ce résultat a une grande répercussion sur la pratique de dépistage, des MG avec une pratique personnelle aussi défaillante seront peu motivé et peu sensibilisé à dépister leurs patientes ou à orienter leurs patientes pour dépistage.

La formation sur la pratique du FCV était très faible, uniquement 5,6% avaient déjà reçu une formation sur la pratique du FCV. Ce qui reste très en dessous des chiffres retrouvés dans une étude tunisienne ou 56% des médecins généralistes ont déjà reçu une formation pratique sur le FCV.

Ce nombre très réduit de médecins formés explique largement la pratique très faible du FCV par les médecins généralistes ou presque la totalité des médecins 92,8% ne l'avaient jamais pratiqué chez leurs patientes et ou le manque de formation était la principale raison de non pratique du FCV chez presque la tous les médecins 95,5%. Ce manque signe l'importance d'une formation organisée dans ce domaine qui doit être prioritaire dans tout programme de dépistage du cancer du col. Cette pratique presque inexistante de FCV est très en dessous des chiffres retrouvés en France ou 79% des MG ont déjà pratiqué un FCV [6] et des chiffres de l’étude tunisienne ou 41,25% MG ont déjà pratiqué un FCV [7]. En Bretagne, 80% des médecins déclaraient réaliser le FCV [18].

Dans une étude canadienne plus de 90% des médecins faisaient un FCV à leurs patientes lors de l'examen périodique [19]. Dans ces études les MG rapportaient plus la réticence des femmes et des problèmes logistiques comme obstacles à la pratique du FCV [6, 7] et non pas le manque de formation comme dans notre étude. Le Maroc, comme la plupart des pays en développement, ne dispose ni des ressources, ni des infrastructures, ni de personnel formé nécessaires pour faire un programme de dépistage par FCV [20] Actuellement l'OMS et la CERVICAL CANCER ACTION préconise d'autres méthodes de dépistage peu couteuse et techniquement simple pouvant remplacer le FCV comme l'Inspection visuelle du col avec l'acide acétique, cette méthode a été choisi comme méthode de dépistage dans le nouveau programme de détection précoce du cancer du col au Maroc [3–21].

Plus de la moitié (52,1%) ne faisaient jamais d'examen vaginal à leurs patientes et quand il était fait 29,4% n'utilisaient pas de speculum. La pratique de l'examen gynécologique était également défaillante dans l’étude ougandaise ou 62% faisaient fréquemment l'examen vaginal et seulement 12% utilisaient le speculum lors de l'examen [9]. Ces résultats impose une correction de la formation initiale pratique du MG qui doit être capable de faire un examen gynécologique correct de la femme en utilisant le speculum avant même d'apprendre les méthodes de dépistage du cancer du col.

A la lumière de nos résultats, une formation initiale adéquate des médecins généralistes aussi bien théorique que pratique parait nécessaire en insistant sur le rôle primordial du MG dans le dépistage du cancer du col.

La formation continue est aussi à développer en organisant des séminaires de formation sur le cancer du col en tenant compte des besoins des MG et des moyens disponibles. Il ne faut pas oublier la sensibilisation des femmes au dépistage qui pourra faciliter les actions ultérieures de prévention et de dépistage dans le cadre du programme national de prévention et de contrôle des cancers au Maroc.

## Conclusion

Nos résultats soulignent clairement la nécessité de mettre en place des actions pour améliorer les connaissances des médecins généralistes théoriques et pratique en ce qui concerne l’épidémiologie, la prévention primaire et le dépistage du cancer du col. Une formation universitaire et continue des médecins généralistes se doit d'intégrer ces notions et doit obligatoirement être améliorée et évaluée. Le nouveau programme de dépistage qui va être mis en place doit tenir compte de ces défaillances et donner au médecin généraliste les moyens nécessaires pour jouer son rôle dans le dépistage tout en définissant clairement son rôle dans le parcours de soins. Il est aussi important d'organiser des compagnes de sensibilisation de la communauté marocaine surtout féminine à l'importance du dépistage du cancer du col pour garantir son adhérence aux programmes de prévention.
